# Impact of Photobiomodulation on the Pro-Osteogenic Activity of Dental Pulp Mesenchymal Stem/Stromal Cells

**DOI:** 10.3390/ijms26178174

**Published:** 2025-08-22

**Authors:** Marcella Rodrigues Ueda Fernandes, Gabriella Teti, Valentina Gatta, Aurora Longhin, Ana Cecilia Corrêa Aranha, Mirella Falconi

**Affiliations:** 1 Special Laboratory of Lasers in Dentistry (LELO), Department of Dentistry, School of Dentistry, University of São Paulo, Av. Prof. Lineu Prestes 2227, São Paulo 05508-000, Brazil; marcellafern@alumni.usp.br (M.R.U.F.); acca@usp.br (A.C.C.A.); 2 Department of Biomedical and Neuromotor Sciences, DIBINEM, University of Bologna, 40100 Bologna, Italy; valentina.gatta6@unibo.it; 3 Department of Medical and Surgical Sciences, DIMEC, University of Bologna, 40100 Bologna, Italy; aurora.longhin2@unibo.it (A.L.); mirella.falconi@unibo.it (M.F.)

**Keywords:** photobiomodulation, infrared irradiation, dental pulp mesenchymal stromal cells, bone remodeling, pro-osteogenic factors

## Abstract

Photobiomodulation (PBM) consists of applying low-level laser light to biological tissues, leading to modulation of cellular functions. PBM has recently gained much attention in the field of regenerative dentistry thanks to its powerful effect on tissue repair and regeneration. Dental pulp mesenchymal stem/stromal cells (DP-MSCs) represent the ideal targets in regenerative dentistry due to their ability to stimulate the regeneration of mineralized and soft tissues and the paracrine factors that they produce. Although there have been several studies evaluating the influence of PBM on DP-MSCs’ regenerative capacity, the results are conflicting, and there are few studies on the influence of PBM on the paracrine factors released by DP-MSCs. Therefore, the aim of this study was to investigate the effect of PBM, using different energy doses of laser irradiation, on the osteogenic capacity of DP-MSCs, focusing on changes in gene expression, mineralizing ability, and release of pro-osteogenic factors. DP-MSCs were irradiated in vitro and differentiated into an osteogenic phenotype. A cell viability assay, alizarin red staining, and TEM analysis were carried out to evaluate the effect of PBM on cell activity, morphology, and mineralization ability. The expression of the main osteogenesis-related markers Runx2, Col1A1, ALP, and BMP was measured to evaluate the influence of PBM on the ability of DP-MSCs to differentiate toward an osteogenic phenotype. The release of IL-6 and IL-8, which are mainly involved in bone remodeling processes, was investigated in the cell medium following PBM irradiation. The results showed a high level of cell viability, suggesting a lack of phototoxicity under the tested conditions. Furthermore, PBM had a significant effect on mineral deposition, IL-6 and IL-8 release, and expression of osteogenic markers. TEM analysis showed intracellular modifications linked mainly to mitochondria, the endoplasmic reticulum, and autophagic vesicles after PBM treatment. These findings demonstrated that the impact of PBM on the osteogenic potential of DP-MSCs is energy dose-dependent, supporting its potential as an effective strategy in regenerative dentistry, particularly for enhancing bone remodeling.

## 1. Introduction

Photobiomodulation (PBM) is a low-energy (nonthermal) laser therapy, which utilizes light from the red/infrared end of the visible spectrum. The therapeutic potential of PBM has been recently tested in several fields such as dermatology and dentistry [1]. As an alternative to non-surgical interventions, PBM has been widely adopted in clinical and home-care settings, with the latter largely driven by the availability of numerous commercial handheld and wearable devices.

The biological mechanisms of PBM are believed to be linked to mitochondrial activity, gene expression, protein synthesis, and inflammatory modulation [2]. It has been reported that PBM can induce proliferation and differentiation of distinct cell lineages, such as fibroblasts, osteoblasts, and osteoclasts, and can also stimulate bone regeneration, revascularization, and collagen synthesis [3,4,5,6]. Furthermore, clinical applications such as tissue regeneration, wound healing, pain, and inflammation modulation have been demonstrated [7,8,9,10]. Although several studies have demonstrated PBM’s ability to modulate cell viability and growth, there are conflicting results; this discrepancy may be due to the variability in the protocols used [11,12]. Nevertheless, the molecular and cellular mechanisms underlying the biological effects of PMB need to be clarified in order to optimize the cellular biomodulation efficacy of PMB treatment. In the present study, we investigated the impact of PBM on the osteogenic differentiation potential of mesenchymal stem/stromal cells (MSCs) by subjecting them to varying energy doses of laser irradiation. The ultimate goals were to identify the optimal energy level that enhances osteogenic activity without inducing phototoxic effects and to elucidate the molecular mechanisms underlying PBM’s effects on MSCs.

Recently, PBM has gained much attention in regenerative dentistry with the aim of restoring, repairing, rejuvenating, and regenerating oral and dental tissues [13]. Regenerative dentistry is mainly focused on the activity of MSCs because of their ability for self-renewal, differentiation, immunomodulatory activity, and secretome release [14]. MSCs can be obtained from different adult tissues, such as bone marrow, adipose tissue, skin, umbilical cord, placenta, brain, and dental-derived stem cells [15,16,17]. Dental pulp tissue of deciduous tooth stem cells (SHEDs), dental pulp mesenchymal stem cells (DP-MSCs), periodontal ligament stem cells (PDLSCs), apical papilla stem cells (SCAPs), and dental follicle progenitor cells (DFPCs) are the main stem cells derived from dental tissues [18]. DP-MSCs have a rapid proliferation rate and the ability to differentiate into odontoblasts, osteoblasts, and other cells, and can generate mineralized tissues [19,20].

Previous studies that described the impact of PBM on DP-MSCs mainly assessed cell viability, cell proliferation, and differentiation abilities and did not report any deleterious effects. A few studies also investigated the impact of PBM on MSC secretome production [21]. The positive effects of using red wavelengths compared to infrared wavelengths have attracted a lot of attention [22]. However, infrared wavelengths are primarily used in in vivo studies. Due to its longer optical penetration depth, it can target deeper tissues [23]. Therefore, further studies are required to establish a definitive understanding of PBM’s effects on DP-MSCs.

Taking into consideration the potential of PBM as an adjunctive stimulatory factor for DP-MSCs and the plethora of conflicting results on PBM’s ability to modulate DP-MSCs, the aim of this study was to analyze the effect of PBM on the osteogenic capacity of DP-MSCs using different doses of laser energy, with a focus on changes in gene expression, mineralizing ability, and release of pro-osteogenic factors. Combining these data with ultrastructural morphological analysis allowed us to hypothesize the mechanisms underpinning PBM’s effects on osteogenic regeneration. To this end, DP-MSCs were exposed to an osteogenic medium with and without infrared laser treatment. A cell viability assay and alizarin red staining were carried out to evaluate cell biocompatibility and mineral deposition. The expressions of the main osteogenic markers osteogenic transcription factor Runt-related transcription factor 2 (RUNX2), the alpha chain of type I collagen (COL1A1), alkaline phosphatase (ALP), and bone morphogenetic protein (BMP) were carried out to evaluate the influence of PBM on osteogenic regeneration. Given the key role of cytokines in bone remodeling, the presence of IL-6 and IL-8 was investigated in the cellular microenvironment following laser irradiation. The ultrastructural morphological changes in DP-MSCs after PBM treatment were assessed using transmission electron microscopy (TEM). The results demonstrated a significant impact of PBM on the osteogenic potential of DP-MSCs, confirming its potential in regenerative dentistry. Moreover, the optimal energy level for enhancing cellular osteogenic activity without inducing phototoxic effects was determined.

## 2. Results

### 2.1. Cell Viability Assay Demonstrated a Lack of Photodamage in Irradiated DP-MSCs

**Figure 1:**
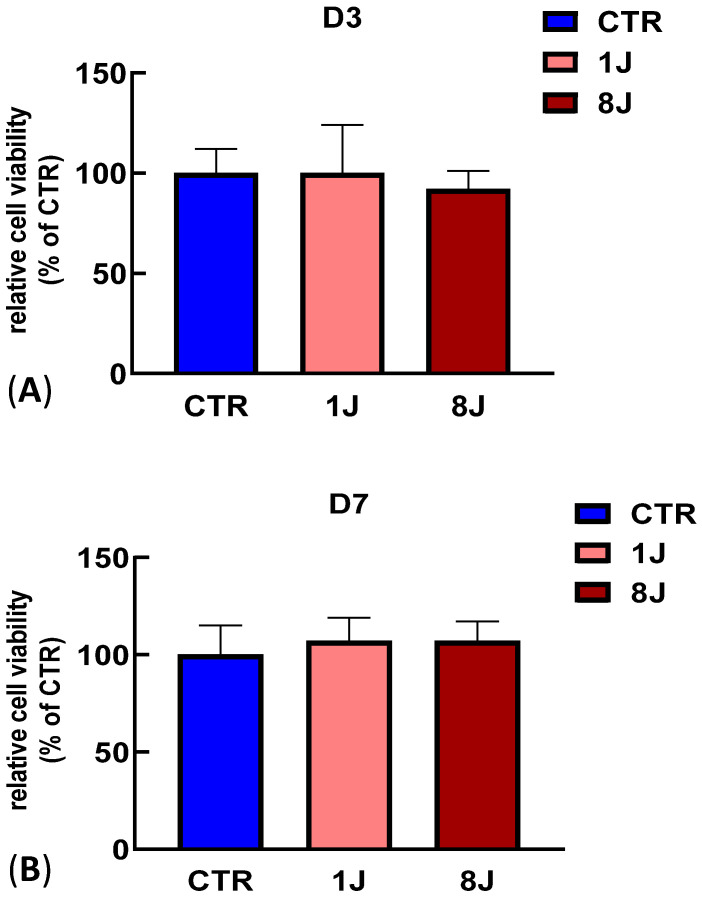
MTT assay showing lack of photodamage from laser irradiation. DP-MSCs irradiated with 1 and 8J of energy for (**A**) 3 (D3) and (**B**) 7 days (D7). MTT assay was performed in triplicate, and the relative quantification is expressed as the mean value ± SD. There were no statistically significant differences compared with CTR (ns).

To demonstrate that the laser irradiation protocol used in this study does not induce photodamage, an MTT assay was carried out on DP-MSCs treated with a laser energy of 1J and 8J for 3 and 7 days. Figure 1 shows a high cell viability after 3 days for cells exposed to 1J and 8J of laser irradiation (Figure 1A). The same results were obtained after 7 days (Figure 1B), confirming that the laser energy utilized in this study does not induce any phototoxicity in DP-MSCs.

### 2.2. Ultrastructural Analysis Demonstrated a Strong Induction of Autophagy by PBM, Which Was Mitigated by Inducing Osteogenic Differentiation

**Figure 2:**
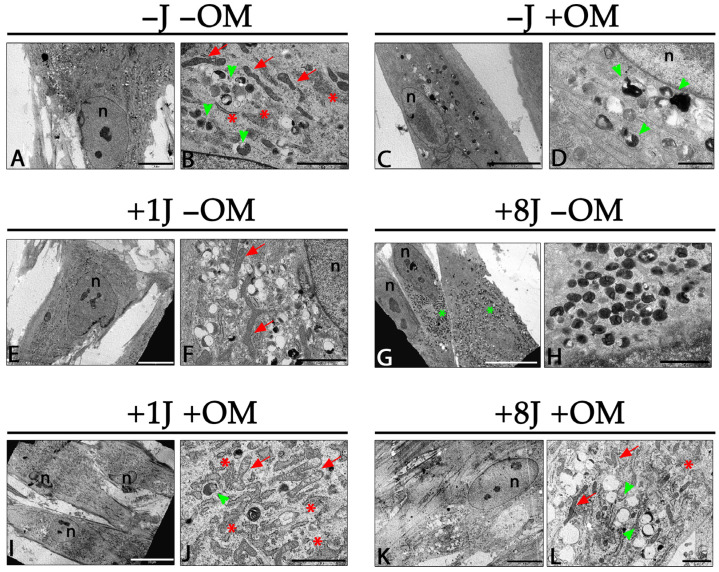
Ultrastructural morphological changes in DP-MSCs induced by PBM irradiation. (**A**) Control DP-MSCs not subjected to PMB treatment or osteogenic induction (−J −OM) (scale bar: 10 µm). (**B**) Magnified image of cytoplasm of −J −OM cells showing long mitochondria (red arrow), developed RER (asterisks), and a few autophagic vesicles (green arrowheads) (scale bar: 2 µm). (**C**) DP-MSCs not subjected to PMB treatment but induced toward osteogenic phenotype for 14 days (−J +OM) (scale bar: 5 µm). (**D**) Magnified image of cytoplasm of −J +OM cells showing a few autophagic vesicles (green arrowheads) (scale bar: 1 µm). (**E**) DP-MSCs irradiated with 1J of energy but not subjected to osteogenic induction for 14 days (+1J −OM) (scale bar: 10 µm). (**F**) Magnified image of cytoplasm of +1J −OM samples showing long mitochondria (red arrows) and an increased number of autophagic vesicles (scale bar: 2 µm). (**G**) DP-MSCs exposed to 8J irradiation and not subjected to osteogenic differentiation (+8J −OM). Cytoplasm is entirely filled with primary and secondary lysosomes (green asterisks) (scale bar: 20 µm). (**H**) Magnified image of cytoplasm of +8J −OM cells showing lysosomes (scale bar: 2 µm). (**I**) DP-MSCs exposed to 1J irradiation and osteogenic differentiation (+1J +OM) (scale bar: 20 µm). (**J**) Magnified image of cytoplasm of +1J +OM cells showing a well-developed RER that occupies entire space (red asterisks). Several mitochondria (red arrows) and a few autophagic vesicles (green arrow heads) were detected (scale bar: 2 µm). (**K**) DP-MSCs exposed to 8J irradiation and osteogenic differentiation (+8J +OM) for 14 days (scale bar: 10 µm). (**L**) Magnified image of the cytoplasm of +8J +OM cells showing regular morphology of cellular organelles such as mitochondria (red arrows), RER (red asterisks), and autophagic vesicles (green arrow heads) as the control cells (scale bar: 2 µm).

TEM ultrastructural analysis was performed to investigate the morphological changes induced by PBM with and without osteogenic induction. Control DP-MSCs (no PBM treatment or osteogenic medium; (−J −OM) showed a spindle-like and polygonal morphology with clear nuclei and nucleoli. In the cytoplasm, several long mitochondria and a well-developed rough endoplasmic reticulum (RER) occupied a significant portion of the cytoplasmic space (Figure 2A,B). Primary and secondary lysosomes were easily detected in the cytoplasm, suggesting an intense turnover of cellular organelles (Figure 2B). Cells treated with the osteogenic medium for 14 days but not given the PBM treatment showed a very similar morphology to that of the control cells except for a reduced number of autophagy vesicles (Figure 2C,D), morphological changes related to the synthesis of osteogenic factors.

DP-MSCs exposed to 1J PBM treatment for 14 days without osteogenic induction maintained a spindle-like polygonal morphology (Figure 2E). Long mitochondria and a developed RER were detected in the cytoplasm (Figure 2F). A higher number of autophagic vesicles was observed compared to samples without PBM treatment (Figure 2F). DP-MSCs exposed to 8J of PBM had a large number of autophagy-related lysosomes (Figure 2G), which almost filled the entire cytoplasmic area. Higher magnification images showed several primary and secondary lysosomes and myelin figures, suggesting a high turnover of cytoplasmic organelles such as mitochondria (Figure 2H). DP-MSCs treated with 1J of laser irradiation in combination with osteogenic induction for 14 days did not show an altered morphology, and autophagic processes were greatly reduced (Figure 2I). The cytoplasm only showed a few secondary lysosomes and was mostly filled with RER and mitochondria (Figure 2J). DP-MSCs exposed to 8J of laser irradiation and osteogenic induction for 14 days exhibited a morphology very similar to that of control cells (Figure 2K). A regular RER, mitochondria, and autophagic vesicles were observed in the cytoplasm (Figure 2L).

### 2.3. Laser Irradiation Enhanced the Ability of DP-MSCs to Differentiate Toward an Osteogenic Phenotype

To investigate the influence of PBM on the expression of the osteogenic markers RUNX2, COL1A1, BMP2, and ALP, real-time PCR was carried out on DP-MSCs irradiated with 1J or 8J of energy and stimulated with osteogenic medium for 3, 7, and 14 days.

**Figure 3:**
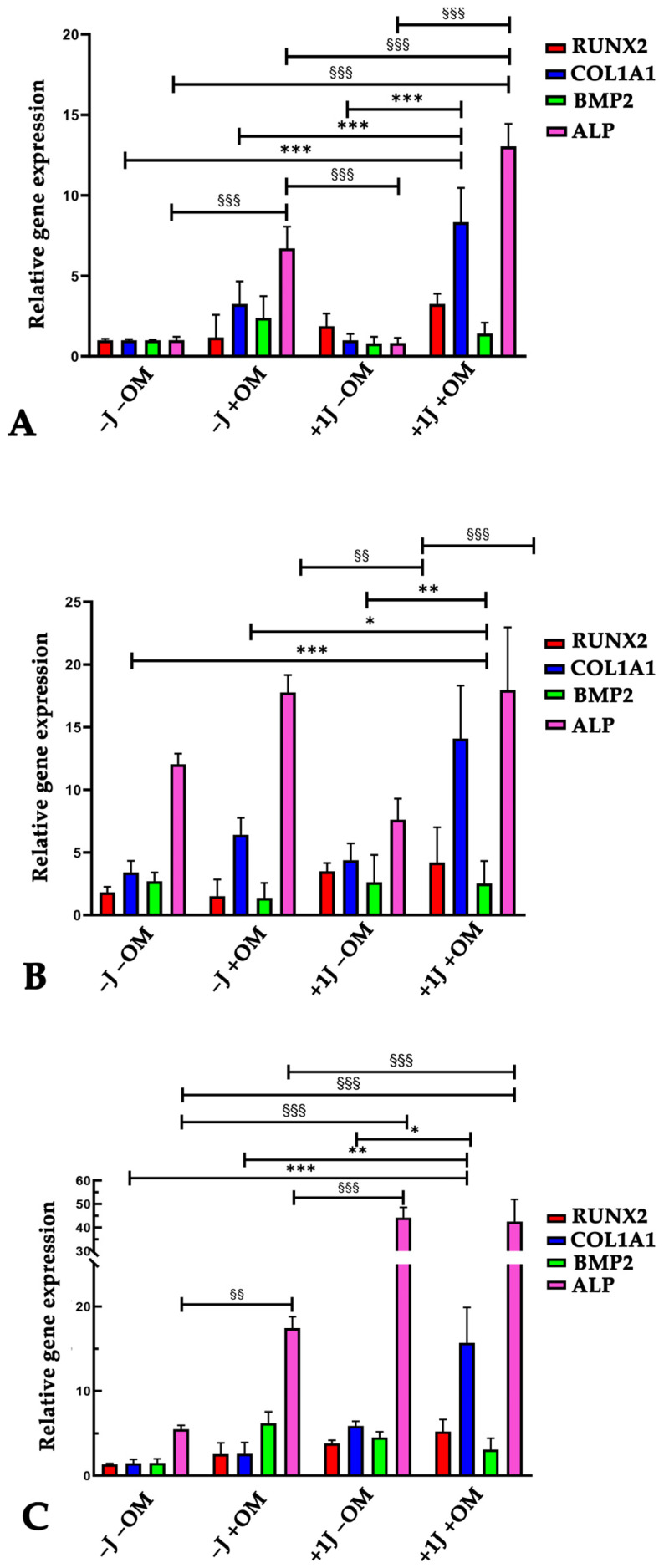
Relative gene expression of osteogenic markers RUNX2, COL1A1, BMP2, and ALP using real-time PCR in DP-MSCs exposed to PBM (±1J) and/or osteogenic medium (±OM) for 3 (**A**), 7 (**B**), and 14 days (**C**). Data are shown as fold increases relative to control group (DP-MSCs not subjected to irradiation or OM; −J −OM) at D3. Results are expressed as the mean ± SD of three independent experiments. * *p* < 0.05, ** *p* < 0.01, and *** *p* < 0.001 for COL1A1 mRNA expression compared to −J −OM samples. ^§§^ *p* < 0.01, and ^§§§^ *p* < 0.001 for ALP mRNA expression compared to −J −OM samples.

There was a gradual upregulation of the osteogenic markers COL1A1 and ALP in the cells subjected to 1J irradiation and osteogenic stimulation (Figure 3A,B), reaching a 15-fold higher COL1A1 expression level and 40-fold higher ALP expression level after 14 days compared to the not irradiated cells and lacking osteogenic induction (Figure 3C). The change in the expression of the transcription factor RUNX2 slightly increased but was still significantly different compared to the untreated samples (−J −OM) (Figure 3A–C), while the expression of the osteogenic marker BMP2 did not show any significant differences compared to the control samples (Figure 3A–C).

**Figure 4:**
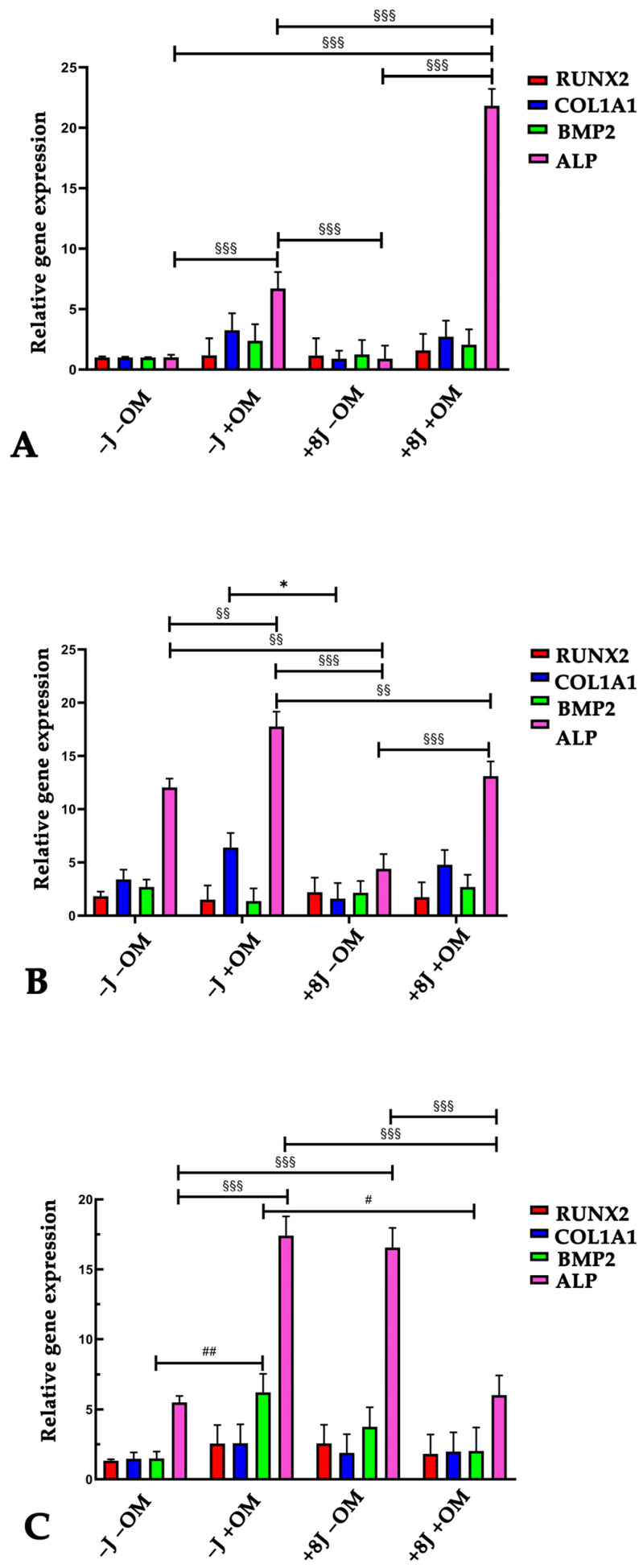
Relative gene expression of osteogenic markers RUNX2, COL1A1, BMP2, and ALP using real-time PCR in DPSCs exposed to PBM (±8J) and/or osteogenic medium (±OM) for 3 (**A**), 7 (**B**), and 14 days (**C**). Data are shown as fold increases relative to control group (DP-MSCs not subjected to irradiation and OM; −J −OM) at D3. Results are expressed as the mean ± SD of three independent experiments. * *p* < 0.05 for COL1A1 mRNA expression compared to −J −OM samples. ^#^ *p* < 0.05, ^##^ *p* < 0.01 for BMP2 mRNA expression compared to −J −OM samples. ^§§^ *p* < 0.01, and ^§§§^ *p* < 0.001 for ALP mRNA expression compared to −J −OM samples.

In the 8J irradiated cells, there was a weaker effect of PBM on osteogenic induction based on the expression levels of all the investigated osteogenic markers (Figure 4A–C). There was a 15-fold increase in ALP expression, while Runx2 and COL1A1 expression slightly increased after 7 days of treatment compared to the control samples (−J −OM) (Figure 4C). Similarly to the 1J irradiated samples, the DP-MSCs irradiated with 8J of energy without osteogenic induction did not show a significant difference in BMP2 expression compared to the control samples even after 14 days of treatment (Figure 4C).

### 2.4. Laser Irradiation Enhanced the Ability of DP-MSCs to Deposit Mineral Crystals

**Figure 5:**
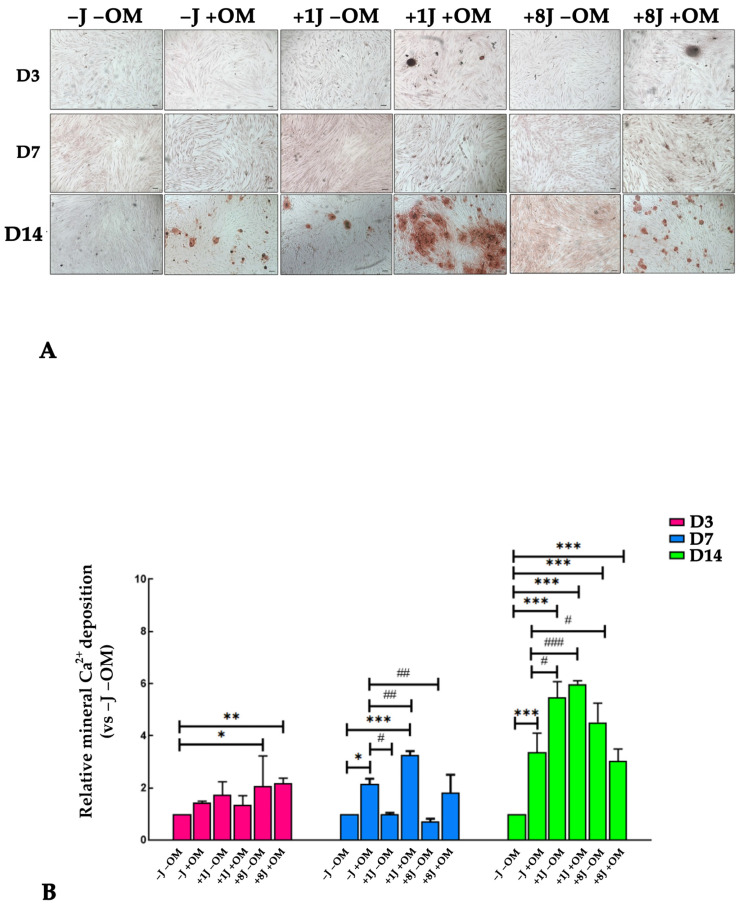
(**A**) Representative images of alizarin red-stained DP-MSCs exposed to 1J oI r 8J of energy irradiation and/or osteogenic medium (OM) for 3 (D3), 7 (D7), and 14 days (D14). (**B**) Quantification of calcium mineral deposition expressed as fold increases relative to untreated samples (−J −OM) whose value was fixed to 1. Results are expressed as the mean ± SD of three independent experiments. * *p* < 0.05, ** *p* < 0.01, and *** *p* < 0.001 compared to −J −OM samples. ^#^ *p* < 0.05, ^##^ *p* < 0.01, and ^###^ *p* < 0.001 compared to −J +OM samples.

To demonstrate the effect of PBM on in vitro mineralization, the DP-MSCs were irradiated and stimulated toward an osteogenic phenotype for 3, 7, and 14 days and then stained with alizarin red to visualize mineral deposition. The amount of red calcium deposition was subsequently quantified using a spectrophotometer. Figure 5 clearly shows the presence of red spots, which correspond to calcium deposits, in the samples stimulated towards an osteogenic phenotype for 14 days. Furthermore, the size and number of mineral deposits were higher in the samples irradiated with 1J (+1J +OM) and 8J (+8J +OM) of energy for 14 days (Figure 5A). Quantitative analysis found that there was a 6-fold increase in mineral deposits in the +1J +OM samples and a 4-fold increase in the +8J +OM samples after 14 days (Figure 5B).

### 2.5. Laser Irradiation Regulated the Release of IL-6 and IL-8 Cytokines During Osteogenic Differentiation

**Figure 6:**
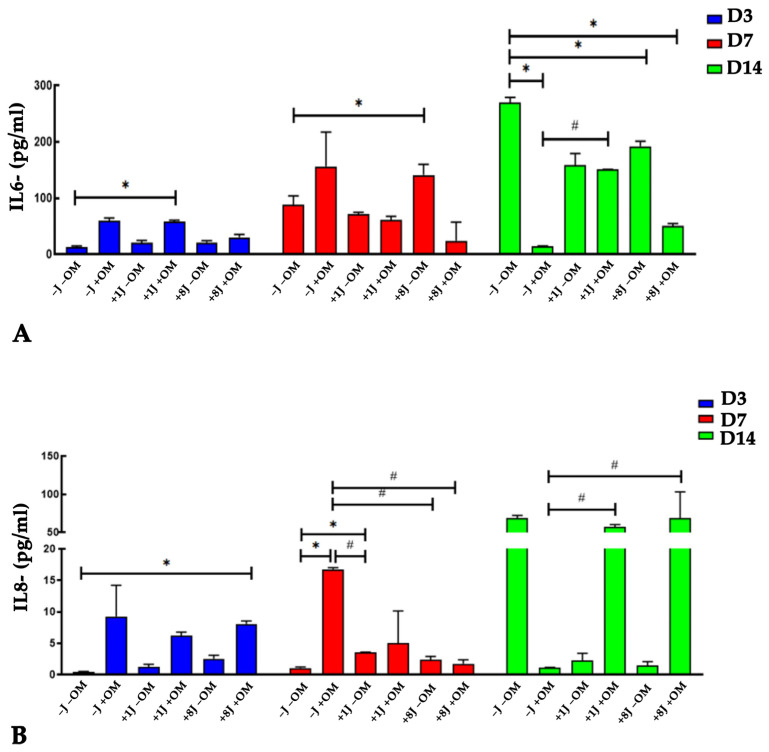
Quantitative analysis of (**A**) IL-6 and (**B**) IL-8 release into cell medium from DP-MSCs after osteogenic differentiation and PBM treatments. Samples were collected and analyzed after 3 (D3), 7 (D7), and 14 (D14) days. Controls consisted of DP-MSCs not subjected to PBM or osteogenic differentiation (−J −OM) or DP-MSCs induced toward an osteogenic phenotype without laser irradiation (−J +OM). Results are expressed as the mean ± SD (pg/mL) of three independent experiments. * *p* < 0.05 compared to −J −OM samples. ^#^ *p* < 0.05 compared to −J +OM samples.

Given the importance of cytokines in bone remodeling, we investigated the release of IL-6 and IL-8 into the microenvironment following PBM treatment. There was a gradual increase in IL-6 and IL-8 secretion from the samples stimulated to undergo osteogenic differentiation for 3 and 7 days without any laser treatment (−J +OM) (Figure 6A,B). IL-6 and IL-8 levels were higher in the +1J −OM and +8J −OM cells, but these levels were lower than those of the differentiated cells not subjected to PBM (−J +OM) (Figure 6A,B). The combination of PBM and osteogenic stimulation (+1J +OM and +8J +OM) for 3 days increased the release of IL-8 to levels similar to those of the −J +OM samples (Figure 6B), while no significant difference was observed after 7 days of treatment (Figure 6B). The IL-6 levels in the +J +OM samples did not show any significant differences compared to the −J −OM and −J +OM groups at 3 and 7 days (Figure 6A,B).

After 14 days of differentiation and laser treatment, the opposite results were observed. The amounts of IL-6 and IL-8 released were higher in the control samples (−J −OM), but they were drastically lower in the −J +OM samples (Figure 6A,B). The cells exposed to 1J and 8J laser irradiation (+1J −OM and +8J −OM) showed a marked increase in IL-6 release, reaching levels slightly below those of the control samples, whereas the amount of IL-8 released was significantly below that of the control samples (Figure 6A,B). The combination of PBM and osteogenic differentiation induction had a strong impact on cytokine release. The concentrations of IL-6 and IL-8 in the 1J +OM and +8J +OM samples rapidly increased to the levels found in the control samples (−L −OM) (Figure 6A,B).

## 3. Discussion

PBM is commonly used in a variety of applications, such as wound healing, reducing inflammation, treating muscle injuries, and bone remodeling [2,24,25,26,27]. It involves the use of therapeutic light, which includes both visible and near-infrared wavelengths of light. The most common light sources used for PBM are lasers and light-emitting diodes (LEDs), which generate biological effects by activating chromophores through the absorption of light at different energy levels [28,29]. Numerous studies have confirmed the positive impact of PBM on the abilities of MSCs, such as enhancing differentiation, proliferation, and migration [3,30,31]. Some studies have demonstrated that PBM therapy boosts the osteogenic and odontogenic differentiation of MSCs, including DP-MSCs [20,32,33,34,35]. However, several limitations hinder the widespread application of PBM in regenerative dentistry, such as the differences in patients’ health conditions and the conflicting results regarding the ability of PBM to modulate DP-MSCs. Additionally, the lack of a standardized and reproducible protocol with consistent parameters that are applicable to all patients undermines the reliability of results and contributes to uncertainty in evaluating the effectiveness of PBM. Therefore, in this study, we investigated the impact of PBM on the osteogenic differentiation potential of DP-MSCs by subjecting them to varying energy doses of laser irradiation. The objective was to identify the optimal energy level that enhanced cellular osteogenic activity without inducing phototoxic effects and to clarify the molecular mechanisms underlying the effects of PBM.

The laser used in this study was a diode laser with a wavelength of 808 nm. The energy dose of 1J was chosen based on findings from the literature [31,32]. Since cell proliferation typically decreases during differentiation, a dose of 2.5 J/cm^2^ was selected to trigger the differentiation process as a low-energy stimulus, particularly in undifferentiated pulp cells, regardless of whether an odontogenic medium was used. The energy dose of 8J (20 J/cm^2^) was chosen to see the possible effects with a higher-energy stimulus. According to the cell viability results, these energies did not induce any phototoxicity in the DP-MSCs, which is in agreement with the scientific literature [36,37,38].

Despite the positive findings indicating the absence of photodamage in the DP-MSCs, we performed TEM analysis to further investigate the cellular ultrastructure following PBM exposure, with particular attention to organelles such as mitochondria. The DP-MSCs exposed to 1J irradiation but not osteogenic induction showed long mitochondria, a developed RER, and autophagy vesicles, all morphological characteristics linked to the self-renewal ability of MSCs [39]. Compared to the control samples, a higher concentration of cellular organelles was observed when the DP-MSCs were exposed to PBM, which indicates that synthesis and degradation of intracellular components were occurring; these processes support the regeneration and repair abilities of MSCs [40]. A high number of autophagic vesicles was observed in DP-MSCs exposed to 8J irradiation, suggesting a strong interplay between autophagy and mitochondria recycling; this crosstalk is critical for preserving MSC function and for addressing age-related decline and enhancing regenerative potential [41]. Since mitochondria are the main targets of PBM, we hypothesize that the 8J treatment triggered a strong cellular reaction to recycle mitochondria, with the aim of preserving the MSCs’ abilities. These morphological features were not observed in the MSCs that were irradiated and induced to undergo osteogenic differentiation. The DP-MSCs treated with 1J and 8J of laser irradiation combined with osteogenic induction for 14 days did not exhibit any changes in morphology and showed a significant reduction in autophagic processes. The cells maintained their spindle and polygonal shape, and the cytoplasm showed a uniform distribution of RER, mitochondria, and autophagic vesicles. These morphological changes are linked to osteogenic processes in which MSCs lose their self-renewal ability to differentiate toward an osteogenic phenotype. PBM irradiation strongly reduces the autophagic processes that support osteogenesis [38].

To better understand the effect of PBM on osteogenic differentiation of DP-MSCs, the expressions of the main osteogenic markers Runx2, COL1A1, BMP2, and ALP were investigated. RUNX2 is a key transcription factor that governs the early stages of osteoblast differentiation, driving MSCs toward the osteoblast lineage and promoting the expression of bone matrix genes, such as type I collagen. Additionally, RUNX2 regulates bone formation by influencing the activity of mature osteoblasts. The expression of RUNX2 was higher in the samples exposed to 1J irradiation and OM, although it was not significantly different compared to control samples (−J −OM). Type I collagen is the most abundant matrix protein and is involved in cell adhesion, proliferation, and the differentiation of osteoblasts. It is produced in stem cells during the early stages of osteogenic differentiation [42,43]. The 1J irradiated cells showed a gradual upregulation of the osteogenic markers COL1A1 and ALP in response to PBM treatment and osteogenic induction, reaching 15-fold higher COL1A1 expression levels and 40-fold higher ALP expression levels after 14 days of stimulation. PBM had a weaker effect on the 8J irradiated cells based on the expression levels of all the investigated osteogenic markers. These data agree with previous studies in which PBM strongly promoted the osteogenic differentiation of human MSCs isolated from different tissues [38,43,44,45]. Furthermore, PBM using 1J of energy had a more pronounced effect on osteogenic differentiation compared to the 8J treatment, suggesting that lower energy levels may be more effective in enhancing the regenerative potential of DP-MSCs.

To confirm the effect of PBM on osteogenic differentiation, the in vitro mineralization ability of DP-MSCs was investigated after PBM treatment and/or bone induction. After 14 days, the samples stimulated toward an osteogenic phenotype showed the presence of red spots, indicating calcium deposits. Both the size and number of mineral deposits were greater in the samples irradiated with 1J (+1J +OM) compared to 8J (+8J +OM). These data support the idea that the mineralization events induced by PBM follow the Arndt–Schulz law: a relatively low radiant exposure promotes cell fate determination, while much higher levels inhibit both proliferation and differentiation. The inhibitory effects of a higher radiant exposure may result from direct interference with chromophore function by photons, or indirect interference through excessive reactive oxygen species production or hyperthermia [46,47,48].

During bone remodeling, several factors are released by the bone to establish a crosstalk between the bone and the tissue microenvironment [49,50]. IL-6 and IL-8 are proinflammatory cytokines that exert a synergistic effect on bone resorption [51]. To support bone remodeling processes, it is crucial to modulate the level of IL-6 and IL-8 in the microenvironment. Since MSCs are known to release immunomodulatory factors, we investigated the potential role of PBM in modulating the secretory capacity of MSCs by measuring the secretion of IL-6 and IL-8.

The PBM treatment reduced the release of IL-6 and IL-8. Combining PBM with inducing osteogenic differentiation attenuated this effect after 14 days of treatment. During bone remodeling, the release of proinflammatory cytokines mainly functions to stimulate osteoclast activation [51]. The finding that PBM reduced the release of IL-6 and IL-8 could result in the favoring of bone synthesis over bone reabsorption.

This study has some limitations, mainly related to the cellular model and the high variability in PBM protocols. Although the human MSCs employed in this study are commercially available, since they are isolated from adult tissues of healthy donors, they exhibit a high degree of donor-dependent variability. Therefore, to properly evaluate the effects of PBM on the osteogenic potential of MSCs, further studies should be performed using cells obtained from multiple donors of different ages and sexes, while also considering any associated pathologies. This information will provide a clearer understanding of the actual effectiveness of PBM in regenerative medicine to support its application in clinical settings.

## 4. Materials and Methods

### 4.1. Human Dental Pulp Mesenchymal Stem/Stromal Cells (DP-MSCs)

DP-MSCs were purchased from Lonza (Euroclone, Milan, Italy) and cultured in Dulbecco’s Modified Eagle Medium (DMEM) (Gibco, Life Technologies, Monza, Italy) supplemented with 10% Fetal Bovine Serum (FBS) (Gibco, Life Technologies, Monza, Italy) at 37 °C in a humidified atmosphere of 5% CO_2_. Confluent cells were sub-cultured once a week using 1% trypsin (Gibco, Life Technologies, Monza, Italy), expanded in new T25 flasks, and maintained at 37 °C in a humidified atmosphere with 5% CO_2_. Cells from the 2nd–5th passages were utilized for the experiments.

### 4.2. PBM Irradiation Protocol and Sample Preparation

DP-MSCs were irradiated using a Laser Duo device (InGaAIP) (MM Optics, São Carlos, Brazil), which contains 1 light-emitting diode that emits infrared light (wavelength: 808 nm). A total energy of 1 J (2.5 J/cm^2^) and 8J (20 J/cm^2^) was delivered (laser beam output area: 0.04 cm^2^; continuous power output: 100 mW ± 20%).

For all the experiments described below, irradiated and non-irradiated cells were seeded into 24-well plates in triplicate at a density of 30^4^ cells/well. The plate cover and the culture medium were removed prior to irradiation to avoid any interference due to light refraction. The laser probe was placed perpendicular to the bottom of the well at a distance of 0.5 cm for 10 s for the 1J treatment and 60 s for the 8J treatment to ensure that all the cells received the radiation. After the treatment, the culture medium was replaced before sample collection was performed 30 min later.

For the osteogenic differentiation experiments, some cells were irradiated and exposed to osteogenic medium or only irradiated. Cells not subjected to laser irradiation and osteogenic differentiation were used as controls. The total number of samples was 8 (n = 8). Table 1 summarizes the experimental groups.

### 4.3. Cell Viability Assay

To demonstrate the lack of any phototoxic damage, a cell viability assay was carried out. The DP-MSCs were not induced toward an osteogenic phenotype. The DP-MSCs were seeded into a 24-well culture plate at a density of 30^4^ cells/well in DMEM supplemented with 10% FBS. After 24 h, the medium was removed, the laser irradiation was performed, and fresh medium was added. The cells were irradiated every day for 3 and 7 days. At the end of each experiment, the medium was changed to fresh medium containing 0.5 mg/mL 3-(4,5-dimethylthiazol-2-yl)-2,5- diphenyltetrazolium bromide (MTT) and left for 2 h at 37 °C. The formazan produced was dissolved in an MTT solvent solution composed of dimethylsulfoxyde/isopropanol (1:1), and the optical density was read at 570 nm (reference wavelength: 690 nm) using a Glomax microplate reader (Promega Italia Srl, Milan, Italy). The results are expressed as a percentage compared to the control samples.

### 4.4. Osteogenic Differentiation

DP-MSCs were seeded into a 24-well culture plate at a density of 30^4^ cells/well in DMEM supplemented with 10% FBS. After 24 h, the medium was replaced with osteogenic medium for 3, 7, and 14 days. The osteogenic medium consisted of alpha MEM medium (Gibco, ThermoFisher Scientific, Milan, Italy) supplemented with 10% FBS, 250 μmol/L ascorbic acid phosphate (Merk Life Science S.r.l., Milan, Italy), 10 mmol/L β-glycerophosphate (Merk Life Science S.r.l., Milan, Italy), 100 nmol/L dexamethasone (Merk Life Science S.r.l., Milan, Italy), and 10 ng/mL transforming growth factor β1 (TGFβ1) (Invitrogen, ThermoFisher Scientific, Milan, Italy). The DP-MSCs were incubated at 37 °C with 5% CO_2_ for 3 (D3), 7 (D7), and 14 (D14) days. DP-MSCs from between the 3rd and 5th passages were used throughout the study.

### 4.5. Transmission Electron Microscopy (TEM)

DP-MSCs were seeded onto cover glasses placed in a 6-well culture plate at a density of 50^4^ cells/well in DMEM supplemented with 10% FBS. After 24 h, the medium was replaced with fresh DMEM or osteogenic medium, and the cells were irradiated as previously described. After 3, 7, and 14 days of treatment, the cells on the cover glasses were fixed with 2.5% glutaraldehyde in a 0.1M cacodylate buffer for 2 h at 4 °C. They were subsequently post-fixed with 1% OsO_4_ in the 0.1 M cacodylate buffer for 1 h at room temperature. After three washes with the 0.15 M cacodylate buffer, the samples were dehydrated in an acetone series (25%, 50%, 70%, 90%, and 100%) and embedded in Epon resin (Merk Life Science S.r.l., Milan, Italy). Sections with a thickness of 100 nm were collected on nickel grids, stained with uranyl acetate and lead citrate, and observed using a Philips CM10 (FEI Company, Eindhoven, The Netherlands). Images were recorded using a Megaview III digital camera (FEI Company, Eindhoven, The Netherlands).

### 4.6. RNA Extraction and Quantitative Real-Time Polymerase Chain Reaction (qRT-PCR)

DP-MSCs were seeded into a 6-well culture plate at a density of 50^4^ cells/well in DMEM supplemented with 10% FBS. After 24 h, the medium was replaced with fresh DMEM or osteogenic medium, and the cells were irradiated as previously described. Samples were collected after 3, 7, and 14 days of treatment, and the total RNA was extracted using a PureLink^TM^ RNA mini kit (Invitrogen, ThermoFisher, Monza, Italy) and quantified using a NanoDrop ND-1000 UV-Vis Spectrophotometer (ThermoFisher Scientific, Monza, Italy). The total RNA (100 ng) was reverse transcribed into first-strand cDNA using the SuperScriptTM III One-Step RT-PCR System (Invitrogen, ThermoFisher Scientific, Monza, Italy). The expression of mRNA was analyzed by quantitative real-time PCR using a 7500 Real-Time PCR instrument (Applied Biosystem, Life Technologies, Monza, Italy). For the analysis, the following TaqMan assays (Applied Biosystems, Life Technologies, Monza, Italy) were used: phosphatase alkaline (ALP Hs01029144_m1), runt-related transcription factor 2 (RUNX2 Hs00231692_m1), collagen type I alpha 1 (COL1A1 Hs00164004_m1), and bone morphogenetic protein 2 (BMP2 Hs00154192_m1). The relative gene expression levels were normalized to the expression of glyceraldehyde 3-phosphate dehydrogenase (GAPDH Hs99999905_m1), and the data are presented as the fold change using the formula 2^−ΔΔCt^, as recommended by the manufacturer (User Bulletin No.2 P/N 4303859, Applied Biosystems). For each experimental time point, the gene expression of treated DP-MSCs was calculated relative to control DP-MSCs (−J −OM at D3). The data are shown as the average of the duplicates ± SD and are representative of the three independent experiments.

### 4.7. Alizarin Red

At the end of the osteogenic differentiation, the deposition of calcium crystals was evaluated using alizarin red S staining at days 3, 7, and 14. Briefly, at the end of the PBM and/or osteogenic differentiation treatment(s), the DP-MSCs were washed with a phosphate-buffered solution (PBS) and fixed in 4% paraformaldehyde in PBS at 4 °C for 20 min. The samples were washed with PBS followed by double deionized water (DDW) and stained with a 1% alizarin red S solution (Merk Life Science S.r.l., Milan, Italy) for 30 min. The stained cells were extensively washed with DDW to remove any non-specific precipitate. Positive red staining represented calcium deposits on the differentiated cells. The experiments were performed in duplicate. Images were acquired using an inverted light microscope (Motic, Europe division, Wetzlar, Germany). The results of the alizarin red S staining assay (Merk Life Science S.r.l., Milan, Italy) were quantified following the manufacturer’s instructions. Briefly, the calcium crystals were dissolved in 10% acetic acid. Then, the samples were incubated at 85 °C for 10 min and centrifuged at 20.000× *g* for 20 min. The supernatant was collected and then neutralized with 10% ammonium hydroxide and the optical density was read at 405 nm using a Glomax microplate reader (Promega Italia Srl, Milan, Italy). The results are expressed as fold changes compared to the control group (−J −OM).

### 4.8. IL-6 and IL-8 Quantification

In order to verify the release of IL-6 and IL-8 following laser irradiation and/or osteogenic differentiation, at the end of each treatment, the cell medium was collected and analyzed using a ProQuantum™ Human IL-6 and IL-8 Immunoassay Kit (Invitrogen, ThermoFisher, Monza, Italy) following the manufacturer’s instructions. The data were analyzed using Proquantum^TM^ software (Invitrogen, ThermoFisher, Monza, Italy) and expressed as absolute values (pg/mL).

### 4.9. Statistical Analysis

Statistical analysis was carried out using GRAPH PAD PRISM 5.0 software (San Diego, CA, USA) by applying one-way ANOVA followed by Tukey’s multiple comparison test. The differences were considered significant at *p* < 0.05.

## 5. Conclusions

In conclusion, our findings support the effectiveness of PBM in enhancing the osteopromoting ability of DP-MSCs without causing cellular damage. PBM could become an important tool for regenerative therapy and tissue engineering.

The combination of PBM and MSCs holds potential as a future modality for hard tissue regenerative dentistry, although the underlying mechanisms of the light–cell interactions are, currently, only partially understood. To obtain more knowledge on the biological effects of PBM, it is crucial to optimize the light parameters for effective delivery, considering the light absorption by the target tissue, while ensuring no harm to the surrounding tissues.

## Figures and Tables

**Table 1:** Experimental groups exposed to (**A**) 1J and (**B**) 8J of laser irradiation and osteogenic medium (OM).

(A)		
Group	1J	OM
1	−	−
2	−	+
3	+	−
4	+	+
(**B**)		
**Group**	**8J**	**OM**
1	−	−
2	−	+
3	+	−
4	+	+

## Data Availability

No new data were created.
